# Allergic inflammation does not impact chemical-induced carcinogenesis in the lungs of mice

**DOI:** 10.1186/1465-9921-11-118

**Published:** 2010-08-26

**Authors:** Konstantinos Doris, Sophia P Karabela, Chrysoula A Kairi, Davina CM Simoes, Charis Roussos, Spyros G Zakynthinos, Ioannis Kalomenidis, Timothy S Blackwell, Georgios T Stathopoulos

**Affiliations:** 1 Applied Biomedical Research & Training Center "Marianthi Simou", Department of Critical Care & Pulmonary Services, General Hospital "Evangelismos", School of Medicine, National and Kapodistrian University of Athens, 3 Ploutarhou Str., 10675 Athens, Greece; 2 2nd Department of Pulmonary Medicine, "Attikon" University Hospital, School of Medicine, National and Kapodistrian University of Athens, 1 Rimini Str.,12462 Haidari, Greece; 3 Division of Allergy, Pulmonary and Critical Care Medicine, School of Medicine, Vanderbilt University, 1161 21st Ave. S, T-1218 MCN, Nashville, TN 37232-2650, USA; 4 Department of Physiology, School of Medicine, University of Patras, Basic Biomedical Sciences Research Building, 2nd Floor, University Campus (Panepistimioupolis), 26504 Rio Patras, Greece

## Abstract

**Background:**

Although the relationship between allergic inflammation and lung carcinogenesis is not clearly defined, several reports suggest an increased incidence of lung cancer in patients with asthma. We aimed at determining the functional impact of allergic inflammation on chemical carcinogenesis in the lungs of mice.

**Methods:**

Balb/c mice received single-dose urethane (1 g/kg at day 0) and two-stage ovalbumin during tumor initiation (sensitization: days -14 and 0; challenge: daily at days 6-12), tumor progression (sensitization: days 70 and 84; challenge: daily at days 90-96), or chronically (sensitization: days -14 and 0; challenge: daily at days 6-12 and thrice weekly thereafter). In addition, interleukin (IL)-5 deficient and wild-type C57BL/6 mice received ten weekly urethane injections. All mice were sacrificed after four months. Primary end-points were number, size, and histology of lung tumors. Secondary end-points were inflammatory cells and mediators in the airspace compartment.

**Results:**

Ovalbumin provoked acute allergic inflammation and chronic remodeling of murine airways, evident by airspace eosinophilia, IL-5 up-regulation, and airspace enlargement. Urethane resulted in formation of atypical alveolar hyperplasias, adenomas, and adenocarcinomas in mouse lungs. Ovalbumin-induced allergic inflammation during tumor initiation, progression, or continuously did not impact the number, size, or histologic distribution of urethane-induced pulmonary neoplastic lesions. In addition, genetic deficiency in IL-5 had no effect on urethane-induced lung tumorigenesis.

**Conclusions:**

Allergic inflammation does not impact chemical-induced carcinogenesis of the airways. These findings suggest that not all types of airway inflammation influence lung carcinogenesis and cast doubt on the idea of a mechanistic link between asthma and lung cancer.

## Introduction

Lung cancer, especially non-small cell lung cancer (NSCLC), presents an epidemic on the rise, accounting for more deaths per year than the next three leading cancers combined [1]. Although smoking cessation is fundamental for lung cancer prevention, currently most lung cancers develop in ex-smokers [2,3]. More importantly, a significant proportion of lung cancers occur in non-smokers and women [4] and there is evidence to support that these cases are governed by a different pathobiology [5]. Hence additional strategies for lung cancer prevention are needed to complement smoking bans, prevention, and cessation [6]. For this to be achieved, better understanding of the molecular pathways that promote airway epithelial carcinogenesis is essential.

Previous work has linked inflammation and carcinogenesis in the gastrointestinal epithelium, and has identified the transcription factor nuclear factor (NF)-*κ*Β as an important tumor promoter [7,8]. We and others have proposed that, in the lungs, carcinogen-induced inflammation and airway epithelial neoplasia are connected via activation of pro-inflammatory NF-*κ*Β [9-11]. However, experimental studies addressing the association of inflammation with lung carcinogenesis have so far focused on innate immune responses, such as those observed in the lungs of heavy smokers and patients with chronic obstructive pulmonary disease [12-15].

Several epidemiologic studies have detected increased incidence of lung cancer in non-smoking patients with asthma [16-20]. The increased risk has been estimated to be 1.5-3.0-fold compared to healthy non-smokers without asthma, while some studies have reported synergy of asthma with female gender, atopy, or polymorphisms in the interleukin (IL)-6 gene towards increasing lung cancer risk [16-20]. One study also found increased risk of dying from lung cancer among patients with asthma [18]. Although observational evidence supports an association of lung cancer with asthma, and although both disease processes have been extensively modeled in mice [9-11,21-26], no study to date has functionally evaluated the effects of the allergic adaptive immune response that characterizes asthma on lung carcinogenesis.

In the present studies, we aimed at determining the impact of experimental-induced allergic airway inflammation on chemical-induced lung carcinogenesis in mice. We hypothesized that either acute or chronic allergic airway inflammation promotes lung carcinogenesis. We chose the most widely used models to emulate the two conditions, the ovalbumin mouse model of allergic respiratory inflammation and the urethane mouse model of lung adenocarcinoma. We used the Balb/c strain of inbred mice, which uniquely displays susceptibility to both compounds. Studies were designed to dissect the effects of allergic airway inflammation on distinct time-periods of tumor initiation and promotion in the respiratory tract. Surprisingly, we found that ovalbumin-induced asthma does not functionally impact urethane-induced lung carcinogenesis.

## Methods

### Reagents

Urethane (ethyl carbamate) was from Sigma Aldrich (St. Louis, MO). Mouse IL-4, IL-5, IL-6, and IL-13 (detection limits: 3.0, 7.0, 5.0, and 1.5 pg/mL, respectively) enzyme-linked immunosorbent assays (ELISA) were from R&D (Minneapolis, MN).

### Animals

Wild-type (*wt*) Balb/c mice were purchased from the Hellenic Pasteur Institute (Athens, Greece) and IL-5 deficient (*il5-/-*) and *wt *(*il5+/+*) mice on a pure C57BL/6 background [27] were purchased from the Jackson Laboratory (Bar Harbor, MN). Animals were inbred at the animal care facilities of the General Hospital Evangelismos (Athens, Greece). All animal care and experimental procedures were approved by the Veterinary Administration Bureau of the Prefecture of Athens, Greece, and conducted according to international standards (http://grants.nih.gov/grants/olaw/GuideBook.pdf). Mice used for experiments were sex-, weight (20-25 g)-, and age (8-10 week)-matched.

### Experimental design

**Figure 1:**
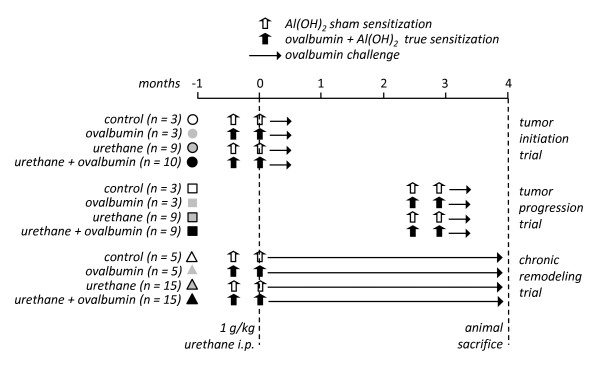
**Experimental design of studies designed to co-model allergic asthma and chemical carcinogenesis in Balb/c mice. *i.p.*, intraperitoneal; *n*, sample size**.

In a first line of experiments, Balb/c mice received a single intraperitoneal (i.p.) injection of urethane (1 g/kg in 100 μl saline) or saline control (100 μl) on experimental day 0. Two-stage ovalbumin treatment composed of an initial sensitization phase [10 μg ovalbumin i.p. in 300 μl Al(OH)_2_] followed by inhaled challenge (10-minute inhalation of aerosolized 50 mg/mL ovalbumin in saline) or sham treatment [sensitization: 300 μl i.p. Al(OH)_2_; challenge: 10-minute inhalation of aerosolized 50 mg/mL ovalbumin in saline] was administered to the same mice in three different protocols: during tumor initiation (tumor initiation trial; sensitization: days -14 and 0; challenge: days 6, 7, 8, 9, 10, 11, and 12), tumor progression (tumor progression trial; sensitization: days 70 and 84; challenge: days 90, 91, 92, 93, 94, 95, and 96), or continuously (chronic remodeling trial; sensitization: days -14 and 0; challenge: days 6, 7, 8, 9, 10, 11, and 12 and thrice weekly thereafter) (Figure 1). Mice were sacrificed after four months. Primary end-points of carcinogenesis were number, size, and histologic type of lung neoplastic lesions (atypical alveolar hyperplasia (AAH), vs adenoma and adenocarcinoma). Secondary end-points of allergic inflammation were inflammatory cells and mediators in bronchoalveolar lavage (BAL), as well as morphologic evidence of airspace enlargement. C57BL/6 *il5+/+ *and *il5-/- *mice received ten consecutive weekly injections of i.p. urethane (1 g/kg in 100 μl saline) [28] and were euthanized after four months. End-point was lung carcinogenesis, as described above.

### Assessment of lung carcinogenesis (primary end-point)

The lungs were explanted after transtracheal inflation with 10% neutral buffered formalin under 25 cmH_2_O pressure. Lung tumors were counted by three blinded readers (KD, SPK, GTS) under a Zeiss Stemi DV4 stereomicroscope at ×12 magnification under both superficial and transillumination modes and averaged per mouse as described previously [9,28,29]. Tumor diameter was determined using microcalipers under stereoscopic vision. Randomly selected tumors were dissected for histologic verification of their adenomatous nature. Excised mouse lungs were fixed in 10% neutral buffered formalin for 24 hours. Lungs were embedded in paraffin based on the lung base, and 5-μm-thick serial transverse sections were cut at three levels of the lungs (apical, median, and basal). Sections were mounted on glass slides and stained with hematoxylin and eosin (H&E). The proportion (percent of total lung lesions) of each type of lung lesions, including AAH, adenoma, and adenocarcinoma were evaluated on the sections from each lung by the above readers and results were averaged per mouse.

### Assessment of lung inflammation (secondary end-point)

BAL was performed using three aliquots of 1000 μl sterile normal saline. BAL volume was not adjusted to body mass, as all mice had similar body mass at harvest. Fluid was combined and centrifuged at 260 g for 10 minutes to separate cells from supernatant. Cells were resuspended in 1 mL phosphate-buffered saline with 1% bovine serum albumin, and total cell count was determined using a grid hemocytometer. Cell differentials were obtained by counting 400 cells on Wright-Giemsa-stained cytocentrifugal specimens. Total cell numbers in BAL were then calculated by multiplying the percentage of each cell type by the total number of cells. IL-4, IL-5, IL-6, and IL-13 were determined in cell-free BAL supernatants by ELISA.

### Statistics

Studies were designed based on power analysis performed online using freely available software (http://www.dssresearch.com/toolkit/sscalc/size_a2.asp). We calculated that, in order to detect 25% differences in the primary end-points of the study with standard deviations of 20% (tumor number and diameter), 95% confidence and 30% statistical power, eight mice per group were needed. All values given represent mean ± standard error of mean. To compare variables between two groups, the Student's t-test or the Mann-Whitney U-test were used for normally and not normally distributed variables, respectively. To compare variables between multiple groups, one-way analysis of variance (ANOVA) with Tukey's post-hoc or Kruskal-Walis with Dunn's post-hoc tests were used for normally and not normally distributed variables, respectively. All probability (*P*) values are two tailed. *P *values < .05 were considered significant. Statistical analyses were performed and graphs were created using Prism Version 5.0 (GraphPad, La Jolla, CA).

## Results

### Combined modeling of allergic inflammation and lung cancer in Balb/c mice using ovalbumin and urethane

**Figure 2:**
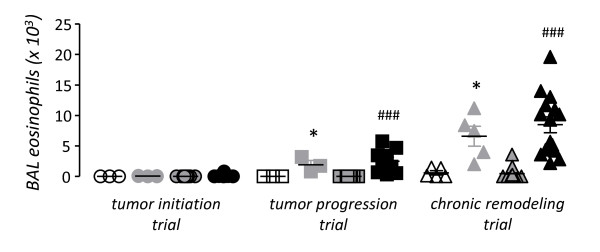
**Bronchoalveolar lavage (BAL) eosinophil numbers in urethane- and ovalbumin-treated Balb/c mice described in Figure 1**. For a legend to the symbols please refer to Figure 1. *Dots*, data points; *lines*, mean; *bars*, standard error of mean. * *P *< 0.05 compared with sham-sensitized mice not treated with urethane; ^### ^*P *< 0.001 compared with sham-sensitized mice treated with urethane.

**Figure 3:**
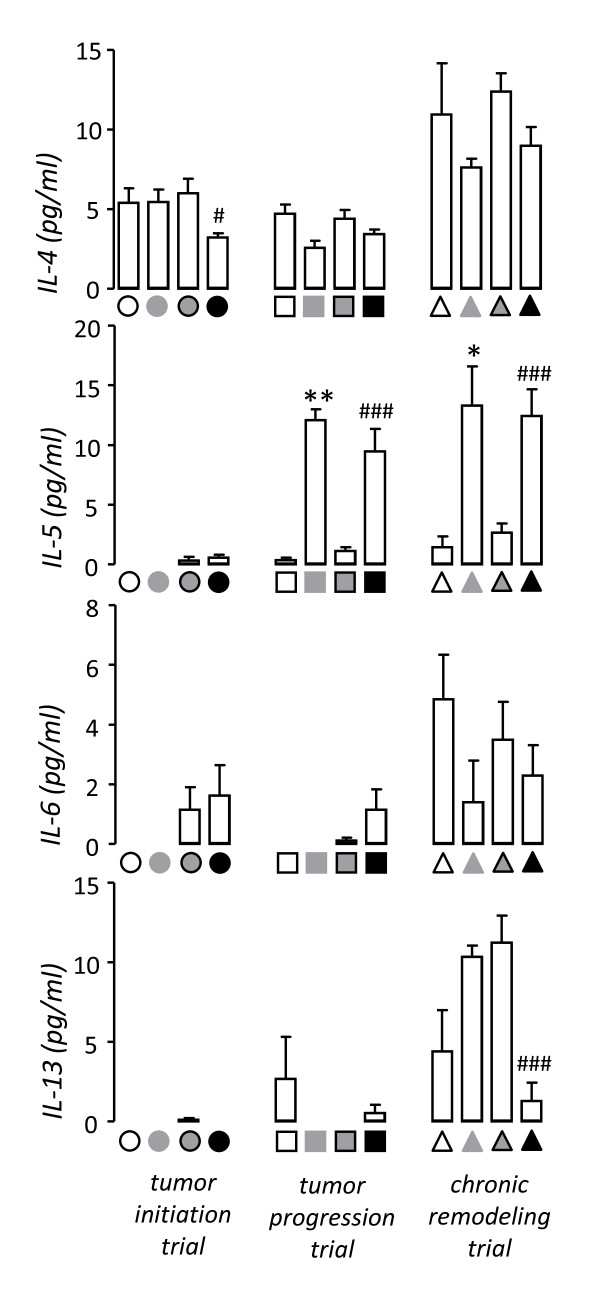
**Levels of interleukins (IL)-4, -5, -6, and 13, as determined by enzyme-linked immunosorbent assay (ELISA) in bronchoalveolar lavage (BAL) of urethane- and ovalbumin-treated Balb/c mice described under Figure 1**. For a legend to the symbols please refer to Figure 1. *Columns*, mean; *bars*, standard error of mean. * *P *< 0.05 and ** *P *< 0.01 compared with sham-sensitized mice not treated with urethane; ^# ^*P *< .05 and ^### ^*P *< .001 compared with sham-sensitized mice treated with urethane.

**Figure 4:**
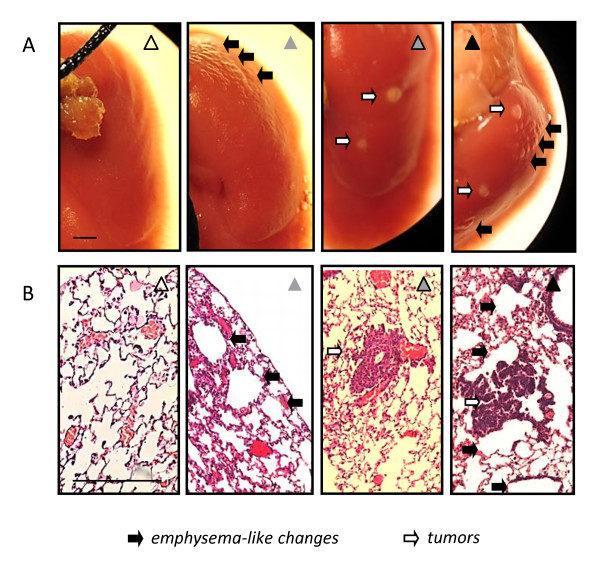
**Macroscopic (*A*; Å = 10) and microscopic (*B*; Å = 40) images of lungs of mice enrolled in the chronic remodeling trial, as described under Figure 1**. For a legend to the symbols please refer to Figure 1. Scale bars = 500 μm.

We initially sought to reproduce ovalbumin-induced allergic airway inflammation and urethane-induced lung carcinogenesis in Balb/c mice, which display sensitivity to both models [9-11,21-26]. For this, mice received a single i.p. dose of urethane or saline control at experimental day 0 (Figure 1). After urethane, carcinogenesis is initiated and promoted during the first four weeks, while thereafter only progression of already established lesions occurs [9,24,26,28]. Hence ovalbumin sensitization and challenge were administered in three different protocols, aiming at induction of allergic airway inflammation during tumor initiation/promotion, during tumor progression, or chronically (Figure 1). Each of the three protocols included appropriate controls for urethane (i.p. saline) and ovalbumin (sham sensitization). After four months, we verified that all mice treated with urethane had lung tumors, while all mice that received saline had no lung tumors. In addition, mice that were sensitized and challenged with ovalbumin displayed increased BAL eosinophils compared with control mice that received sham sensitization, except from mice enrolled in the initiation trial, which had no evidence of BAL eosinophilia since three months had elapsed since ovalbumin challenge (Figure 2). There were no differences between experimental groups in other inflammatory cell types found in BAL, such as macrophages and neutrophils (Table 1). We next examined IL-4, IL-5, IL-6, and IL-13 levels in BAL, since IL-4, IL-5, and IL-13 are major mediators of allergic inflammation [30] and IL-6 gene polymorphisms have been associated with increased lung cancer risk in asthmatics [19]. All mice that were sensitized/challenged with ovalbumin consistently displayed increased BAL IL-5 levels compared with control mice. Again, mice enrolled in the initiation trial did not display increased BAL IL-5, since three months had elapsed from ovalbumin (Figure 3). None of the other cytokines determined (IL-4, IL-6, and IL-13) was consistently increased in association with either ovalbumin or urethane treatments. In addition to increased eosinophil numbers and IL-5 expression in the airspace compartment, we found additional evidence of the effectiveness of chronic ovalbumin challenge in sensitized mice: mice that received prolonged ovalbumin treatment developed airway remodeling as evidenced by macroscopic and microscopic airspace enlargement consistent with dynamic air trapping, a phenotype not encountered in mice that received sham sensitization (Figure 4). The above determinations confirmed that we could effectively model both allergic inflammation and chemical carcinogenesis in the lungs of our experimental mice on the Balb/c background.

**Table 1:** Inflammatory cells (× 10^3^) in bronchoalveolar lavage of urethane- and ovalbumin-treated Balb/c mice described in Figure 1.

	Macrophages	Lymphocytes	Neutrophils	Eosinophils
Tumor initiation trial				

Control	60 ± 25	4 ± 2	1 ± 1	0 ± 0

Ovalbumin	50 ± 20	3 ± 1	1 ± 0	0 ± 0

Urethane	89 ± 14	8 ± 2	1 ± 0	0 ± 0

Urethane + ovalbumin	88 ± 10	7 ± 1	2 ± 1	0 ± 0

Tumor progression trial				

Control	60 ± 18	7 ± 4	1 ± 0	0 ± 0

Ovalbumin	40 ± 33	7 ± 2	4 ± 3	2 ± 1*

Urethane	101 ± 14	13 ± 5	2 ± 1	0 ± 0

Urethane + ovalbumin	49 ± 24	7 ± 2	5 ± 3	2 ± 1###

Chronic remodeling trial				

Control	54 ± 16	7 ± 3	1 ± 1	1 ± 0

Ovalbumin	55 ± 18	8 ± 1	3 ± 1	7 ± 2*

Urethane	47 ± 12	3 ± 1	1 ± 0	0 ± 0

Urethane + ovalbumin	84 ± 19	13 ± 6	7 ± 5	8 ± 1###

* *P *< 0.05 compared with sham-sensitized mice not treated with urethane; ### *P *< .001 compared with sham-sensitized mice treated with urethane.

### Allergic airway inflammation does not impact chemical lung carcinogenesis

**Figure 5:**
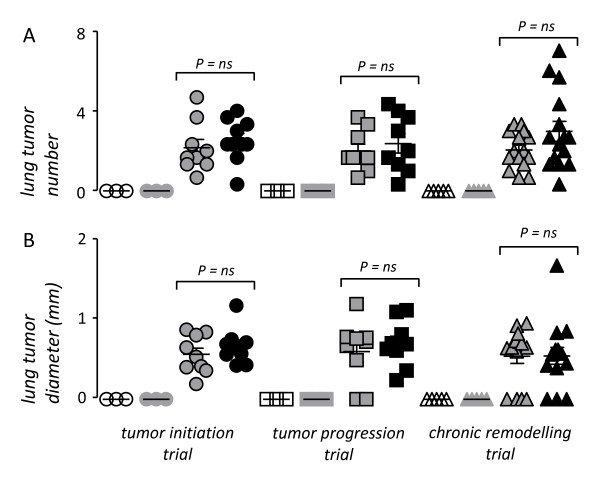
**Parameters of lung carcinogenesis in urethane- and ovalbumin-treated Balb/c mice described under Figure 1**. For a legend to the symbols please refer to Figure 1. *Dots*, data points; *lines*, mean; *bars*, standard error of mean. *ns*, not significant; *P*, probability.

We subsequently assessed lung carcinogenesis, the main end-point of the present study in the above-described Balb/c experimental mice that developed allergic airway inflammation at some point during the multi-stage process of chemical-induced lung carcinogenesis. In contrast to our hypothesis, urethane-treated mice developed equal numbers of lung tumors, irrespective of whether they received ovalbumin or sham sensitization (Figure 5A). The same was true for lung tumor size, which was not affected by the induction of acute or chronic allergic airway inflammation (Figure 5B). In addition, the distribution of lung neoplastic lesions between early (AAH) and more progressed (adenoma, adenocarcinoma) histologic types was not affected by ovalbumin-induced respiratory inflammation. In the tumor initiation trial, sham-sensitized urethane-treated mice had lung neoplastic lesions composed of 84 ± 4% AAH, 13 ± 4% adenomas, and 3 ± 1% adenocarcinomas, while ovalbumin-sensitized urethane-treated mice had 83 ± 3% AAH, 12 ± 2% adenomas, and 5 ± 2% adenocarcinomas (*P *> 0.05); in the tumor progression trial, sham-sensitized urethane-treated mice had lung neoplastic lesions composed of 84 ± 7% AAH, 10 ± 6% adenomas, and 6 ± 2% adenocarcinomas, while ovalbumin-sensitized urethane-treated mice had 84 ± 4% AAH, 11 ± 4% adenomas, and 5 ± 2% adenocarcinomas (*P *> 0.05); finally, in the chronic remodeling trial, sham-sensitized urethane-treated mice had lung neoplastic lesions composed of 79 ± 3% AAH, 16 ± 2% adenomas, and 5 ± 1% adenocarcinomas, while ovalbumin-sensitized urethane-treated mice had 78 ± 2% AAH, 15 ± 2% adenomas, and 7 ± 1% adenocarcinomas (*P *> 0.05). Even mice that received ovalbumin challenge throughout the whole time-course of chemical-induced lung carcinogenesis (chronic remodeling trial) and developed marked allergic inflammation and airway remodeling accompanied by significant air trapping, did not exhibit evidence of enhanced tumor formation or progression. Collectively, these results indicated that allergic inflammation does not mechanistically impact lung carcinogenesis in mice.

### IL-5 does not affect lung carcinogenesis

**Figure 6:**
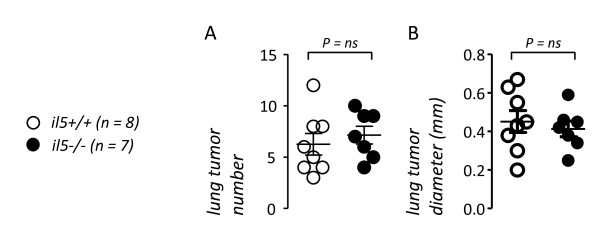
**Lung carcinogenesis in *wild-type *(*il5+/+*) and interleukin (IL)-5 deficient (*il5-/-*) C57BL/6 mice induced by ten weekly doses of urethane after four months latency**. *Dots*, data points; *lines*, mean; *bars*, standard error of mean. *ns*, not significant; *P*, probability.

To further corroborate these negative results, we used mice with genetic deficiency in IL-5 (*il5-/-*), a critical mediator of asthma which was consistently up-regulated in the airspace compartment of mice treated with ovalbumin. For this, wt *il5+/+ *and *il5-/- *mice on the C57BL/6 background received ten weekly doses of urethane and were euthanized after 4 months. *il5+/+ *and *il5-/- *mice developed similar lung tumor numbers of equal size (Figure 6). In addition, lung tumors from *il5+/+ *and *il5-/- *mice had a similar histologic distribution. In specific, *il5+/+ *mice had lung neoplastic lesions composed of 69 ± 5% AAH, 22 ± 4% adenomas, and 9 ± 2% adenocarcinomas, while *il5-/- *mice had 75 ± 3% AAH, 19 ± 2% adenomas, and 6 ± 1% adenocarcinomas (*P *> 0.05). Hence, in addition to ovalbumin-induced allergic inflammation, IL-5, a central mediator of allergic inflammation of the airways, does not influence chemical lung carcinogenesis induced by a prototype carcinogen.

## Discussion

In the present studies we experimentally tested the hypothesis that allergic airway inflammation, such as that observed in asthma, promotes lung carcinogenesis. For this, we generated both ovalbumin-induced allergic inflammation and urethane-induced carcinogenesis in the lungs of Balb/c mice, sensitive to both compounds. Allergic inflammation was induced in both an acute and a chronic fashion and studies were designed for induction of respiratory allergy during distinct time-periods of multi-stage lung carcinogenesis. In stark contrast to what we anticipated based on reports of increased lung cancer incidence in asthmatic humans, we found no evidence that allergic inflammation influences chemical carcinogenesis in the murine lung. This was the case during both tumor initiation and tumor progression in the respiratory tract. Even animals with long-standing allergic airway inflammation resulting in marked structural alterations of pulmonary airways and parenchyma did not display evidence of enhanced tumor formation or progression. These results were furthered by studies on mice genetically engineered to lack IL-5, a mediator of asthma consistently up-regulated in our mice with ovalbumin-induced allergic respiratory inflammation. These mice did not exhibit any difference in lung tumor induction by urethane compared to *wt *littermates. Collectively, these studies indicate that allergic airway inflammation does not functionally affect chemical-induced lung carcinogenesis; that not all types of airway inflammation influence lung carcinogenesis; and that a mechanistic link between asthma and lung cancer may not exist.

Most cases of lung cancer are caused by smoking [31]. In addition to genetic damage, smoking provokes chronic inflammation in the lungs, represented by the spectrum of illness coined chronic obstructive pulmonary disease (COPD) [32]. Multiple lines of evidence from humans, cell and mouse models support that, in addition to the mutational stress imposed by tobacco carcinogens, chronic inflammation caused by smoking and/or in the context of COPD [12] can induce or promote lung cancer formation and progression [33]. In this regard, observations of increased lung cancer incidence in smokers with COPD compared with smokers without COPD after correction for smoking intensity and duration [34,35] have been coupled with functional studies in animal models that have identified and validated candidate molecular culprits for this link, including NF-κΒ, tumor-related protein 53(TRP53, P53), and Janus kinase (JNK) [9-11]. These lines of evidence have established an association between innate immune responses in the lungs and lung carcinogenesis.

However, not all cases of lung cancer are caused by smoking. An estimated 10-15% of lung cancers is attributed to other genetic and environmental factors, such as occupational or domestic exposure to gases, fumes, or irritants and inherited somatic mutations or genetic polymorphisms [36,37]. In this regard, development of adenocarcinomas in never-smoking women in south-east Asia has been the focus of debate [4], and there is evidence to support that these cases are governed by a different pathobiology [5]. In addition, not all inflammatory lung disorders are smoking-related. Importantly, miscellaneous inflammatory and fibrotic pulmonary conditions like pulmonary fibrosis, tuberculosis, or asthma have been reported to be associated with increased lung cancer incidence [36,37]. Several reports now have linked asthma with increased lung cancer incidence [16-20], setting the question of whether allergic airway inflammation promotes carcinogenesis in the respiratory tract.

In an effort to address this issue, we functionally modeled both asthma and chemical-induced lung cancer in mice. We used the most widely available models for this and set power analysis-based criteria to design this work [9-11,21-26]. Evidence for effective induction of asthma-like allergic airway inflammation was sought: ovalbumin-treated mice developed marked airspace eosinophilia and IL-5 up-regulation, widely used biomarkers of asthma [38]. In addition, mice chronically exposed to the allergen developed structural changes reminiscent of the chronic airway remodeling that occurs in humans with difficult-to-treat asthma [23,38]. Despite the above efforts to discover a possible impact of experimental allergic airway inflammation on chemical carcinogen-induced tumor initiation or progression in the lungs of mice, our results show the absence of such an effect. In addition, we found that genetic deficiency in IL-5, a central mediator of allergy and asthma, has no impact on urethane-induced adenocarcinoma formation. This stands in contrast to previous observations from our group on the role of the cytokine in adenocarcinoma progression, in the forms of malignant pleural effusion [39] and metastasis (unpublished data). In fact, we have observed a marked role of IL-5 in promoting intravenous and intrapleural tumor progression via immunomodulatory effects on the host response to tumor. These different results collectively indicate that the effects of IL-5 on malignant effusion and metastasis are specific and do not apply to more early stages of tumor induction, and that different components of the host immune system are involved during the different phases of tumor formation and progression in the respiratory tract.

Inflammation has been linked with cancer formation and progression. However, in contrast to a generalized effect of any type of inflammation on cancer formation, it is more probable that specific cellular, humoral, and transcriptional components of inflammation are involved in lung cancer formation and progression. In the lungs, while tobacco smoke [11] and bacterial product-induced [40] inflammation promote carcinogenesis, our study shows that allergic inflammation characterized by specific induction of eosinophil and IL-5 accumulation does not enhance chemical carcinogenesis. In this regard, while macrophages and neutrophils can function as potent promoters of tumor progression [41,42], eosinophils are probably mere bystanders recruited to tumor sites of necrosis [43]. In addition, while mediators of innate inflammation positioned within the NF-κΒ pathway, such as tumor necrosis factor, promote lung carcinogenesis [7-11,29], our studies provide evidence that inflammatory mediators involved in other inflammatory signaling pathways, such as IL-5, do not affect lung tumor formation and progression.

The shortcomings of our studies are not to be overlooked. We only modeled allergic airway inflammation and chemical lung carcinogenesis using Balb/c mice, a single allergen, and a single carcinogen. In addition, we used the resistant C57BL/6 strain to study the role of IL-5 in lung carcinogenesis. However, Balb/c mice developed both allergic inflammation in response to ovalbumin and lung tumors in response to urethane, and should thus be an appropriate model for the study of the interactions between the two conditions. Moreover, sufficient lung tumors were induced in C57BL/6 mice by multiple urethane doses, facilitating the study of the role of IL-5 in lung tumor formation.

Since the original induction of urethane-induced lung tumors in C57BL/6 mice [28], another group [44] and we have observed increased tumor numbers in the lungs of urethane-treated C57BL/6 mice. This phenomenon could be ascribed to background strain variation, urethane batch variation, or other unidentified reasons. There is no evidence that C57BL/6 mice are currently more sensitive to other commonly used carcinogens, such as 3'-methylcholanthrene, since the original report by Miller et al [28]. Although we and others have observed higher tumor numbers than Miller et al., C57BL/6 mice are still highly resistant to urethane-induced lung tumorigenesis.

Although negative, our study holds value in streamlining future research [45,46]. Our negative findings may aid in focusing future basic investigations into the relationship of lung carcinogenesis with inflammation in pertinent directions. In addition, the proposed absence of a mechanistic impact of allergic inflammation on lung carcinogenesis may aid towards focusing on other possible explanations for increased lung cancer detection in patients with asthma [16-20], such as increased medical surveillance of this patient population. Another possible explanation for the human epidemiologic studies showing increased cancer detection in asthmatics is non-reported (occult) smoking in self-reported non-smokers, since these studies did not employ tobacco exposure biomarker assessment, such as cotinine or carboxyhemoglobin.

## Conclusions

We showed herein that allergic airway inflammation of mice that is similar to human bronchial asthma does not affect tumor initiation or progression in the respiratory tract triggered by a prototype chemical carcinogen. These unexpectedly negative results may aid in the future in better understanding the increased lung cancer risk observed in humans with asthma.

## Competing interests

The authors declare that they have no competing interests.

## Authors' contributions

KD carried out mouse experiments and immunoassays, performed histologic analyses, and helped to draft the manuscript. SPK participated in mouse experiments and immunoassays, performed histology, and helped to draft the manuscript. CAK participated in mouse experiments and helped to draft the manuscript. DCMS participated in the design of the study and helped to draft the manuscript. CR, SGZ, IK, and TSB participated in the design of the study helped to draft the manuscript. GTS conceived and designed and coordinated the study, carried out mouse experiments, performed histologic analyses, analyzed the data, wrote the manuscript, and revised the paper after peer-review. All authors read and approved the final manuscript.
